# Stakeholders' perspectives on research integrity training practices: a qualitative study

**DOI:** 10.1186/s12910-021-00637-z

**Published:** 2021-05-28

**Authors:** Daniel Pizzolato, Kris Dierickx

**Affiliations:** Department of Public Health and Primary Care, Centre for Biomedical Ethics and Law, KU Leuven, 3000 Leuven, Belgium

**Keywords:** RI training practices, RI education, Qualitative study, Preventive measures, Virtue-related training

## Abstract

**Background:**

Even though research integrity (RI) training programs have been developed in the last decades, it is argued that current training practices are not always able to increase RI-related awareness within the scientific community. Defining and understanding the capacities and lacunas of existing RI training are becoming extremely important for developing up-to-date educational practices to tackle present-day challenges. Recommendations on how to implement RI education have been primarily made by selected people with specific RI-related expertise. Those recommendations were developed mainly without consulting a broader audience with no specific RI expertise. Moreover, the academic literature lacks qualitative studies on RI training practices. For these reasons, performing in-depth focus groups with non-RI expert stakeholders are of a primary necessity to understand and outline how RI education should be implemented.

**Methods:**

In this qualitative analysis, different focus groups were conducted to examine stakeholders’ perspectives on RI training practices. Five stakeholders' groups, namely publishers and peer reviewers, researchers on RI, RI trainers, PhDs and postdoctoral researchers, and research administrators working within academia, have been identified to have a broader overview of state of the art.

**Results:**

A total of 39 participants participated in five focus group sessions. Eight training-related themes were highlighted during the focus group discussions. The training goals, timing and frequency, customisation, format and teaching approach, mentoring, compulsoriness, certification and evaluation, and RI-related responsibilities were discussed. Although confirming what was already proposed by research integrity experts in terms of timing, frequency, duration, and target audience in organising RI education, participants proposed other possible implementations strategies concerning the teaching approach, researchers' obligations, and development an evaluation-certification system.

**Conclusions:**

This research aims to be a starting point for a better understanding of necessary, definitive, and consistent ways of structuring RI education. The research gives an overview of what has to be considered needed in planning RI training sessions regarding objectives, organisation, and teaching approach.

**Supplementary Information:**

The online version contains supplementary material available at 10.1186/s12910-021-00637-z.

## Background

The production of credible and reproducible science is considered a crucial element across disciplines [1–3]. Failing to provide reproducible research depends on different factors, namely, researchers’ irresponsible conduct, sloppy practices, and the lack of knowledge [4–6]. Carrying out reliable and trustworthy science is possible by doing research in line with the standards of research integrity (RI). RI is defined as performing research in adherence to responsible research practices, and in line with high ethical, methodological and professional standards [7]. Responsible research practices and RI principles are highlighted in the European Code of Conduct for Research Integrity (ALLEA revised edition 2017) [8].

Promoting RI within the research environment, at individual and collective levels, can be done by developing precise guidelines, undergoing the publication review process, permanent data sharing and by implementing preventive measures [9]. In addition to the measures mentioned above, RI education has been indicated as an important element for developing and fostering RI culture. The importance of organising "appropriate and adequate training in ethics and RI at the institutional level" is highlighted in the ALLEA code [8]. As part of the research training, providing adequate RI education is also underlined in several other sources and European national guidance [8, 10–13]. However, there are no indications about how the training should be carried out. Furthermore, across Europe, there is neither harmonisation on the structure, timing, or contents of such educational programs nor a standard view about the purpose that leads to the development of this training [14].

The majority of the recommendations on implementing and structure RI education have been made by selected stakeholders involved in (inter)national working groups on RI with specific RI-related expertise [9–12]. However, a limited number of qualitative studies explore the existing situation, lacunas and future needs of RI education [15, 16].

This qualitative study aims to better understand key stakeholders' perspectives, including non-RI experts, on how RI education should be implemented.

## Methods

The study follows the Consolidated Criteria for Reporting qualitative research (COREQ) checklist for focus groups [17].

The methods section is divided into the following sub-sections: focus group organisation, data collection, participants, data analysis, and ethics.

### Focus groups organisation

To gain diverse perspectives regarding current RI training practices, we identified five different stakeholders' groups: publishers and peer reviewers, researchers on RI, RI trainers, PhDs and postdoctoral researchers, and research administrators working within academia. Except for the focus group organised with PhD students and postdocs, we looked for precise events involving each time one of the stakeholders’ groups to organise a specific focus groups. After having identified precise meetings/events and having obtained the organisers' consent, we sent them the informational leaflet containing the purpose of the study and all needed information related to it, asking them to disseminate it amongst the event's participants for the recruiting phase (Table 1-focus group organisation). The recruitment of the participants was done during the events helped by the event’s organisers, and the focus groups were carried out at the end of the meetings.

**Table 1:** Focus groups organisation

Focus group	Context in which was organized the focus groups (Country)	Topic of the event
Publishers and peer-reviewers	Peere meeting (https://www.peere.org/) (London, UK)	New frontiers of peer review
Researchers on RI	VIRT^2^UE consortium meeting (https://cordis.europa.eu/project/id/787580) (Oslo, Norway)	Consortium meeting of the EU funded project VIRT^2^UE
RI trainers	Train-the-trainer program (https://oeawi.at/en/training-train-the-trainer/) (Vienna, Austria)	Research integrity train-the-trainer program organized by the Austrian agency for RI
PhDs and postdoctoral researchers	KU Leuven (Leuven, Belgium)	Specifically organized with KU Leuven doctoral candidates and postdoctoral researchers
Research administrators	EARMA general meeting (https://www.earma.org/) (Bologna, Italy)	General assembly organized by the European Association of Research Managers and Administrators

### Data collection

We performed five focus groups between October 2018 and March 2019. Each focus group involved specific stakeholders’ groups (Table 1-focus group organisation). The first researcher moderated the focus groups while another researcher acted as an observer. The focus groups lasted around one hour, engaging six to ten participants each time (Table 2-participants’ characteristics). Each focus group was carried out with no less than six participants, following the recommendation that a group of at least six participants is needed to gain valuable results [18].

**Table 2:** Participants’ characteristics

Focus group (number of participants)	Disciplines of participants	Profiles of the participants	Training in which participants were involved (number of participants)
Publisher and peer-reviewers (9)	Social sciences Computer sciences Biomedical sciences Applied sciences	Academic researchers Professors Journal editors Researcher for a private company	No (6) Online training as a trainee (1) In-person training as a trainee (2)
Researchers on RI (10)	Biomedical sciences Humanities Social sciences Natural sciences	Academic researchers Professors RI officers RE committee	In-person training as a trainee (8) In-person training as a trainer (2) In-person training as a trainee or a trainer (4)
RI future trainers (7)	Biomedical sciences Natural sciences Applied sciences Social sciences	Academic researchers Professors RI officers Journal editors	In-person training as a trainee (7)
PhDs and postdoctoral researchers (6)	Humanities Social sciences Biomedical sciences	PhD researchers Postdoc researchers	In-person training as a trainee (5) No (1)
Research administrators (7)	Natural sciences Biomedical sciences Social sciences Applied sciences	Research administrators RE committee RI officers	Online training as a trainee (1) In-person training as a trainee (3) In-person training as a trainer (1) No (2)

Before starting the focus groups, participants were asked for demographic information and information about their ongoing and previous involvement in RI-related education and training sessions (Additional file 1: Demographic Characteristics). Moreover, before the beginning of the focus group, we have foreseen some times in which participants were able to ask for clarifications regarding the focus group itself and its content.

Each focus group was conducted using a self-developed guideline (Additional file 2: Interview guideline). We posed open-ended RI training-related questions amongst the participants to raise discussions. We structured our interview guide starting with two general introductory questions about the topic. Afterwards, we divided our guideline into four different sections to probe specific themes more in-depth.

Each focus group was audio-recorded and behavioural observations were noted. The first researcher transcribed the recordings verbatim.

### Participants

Thirty-nine participants from the five different groups agreed to participate (Table 2-participants’ characteristics). Participants have different discipline-related backgrounds ranging from biomedical sciences to humanities and different profiles ranging from being professors and academic researchers to being research administrators or being part of research ethics committees. Regarding participants having a researcher’s profile, their career level and related experience ranged from doctoral students to senior researchers at the time of the focus groups.

**Fig. 1:**
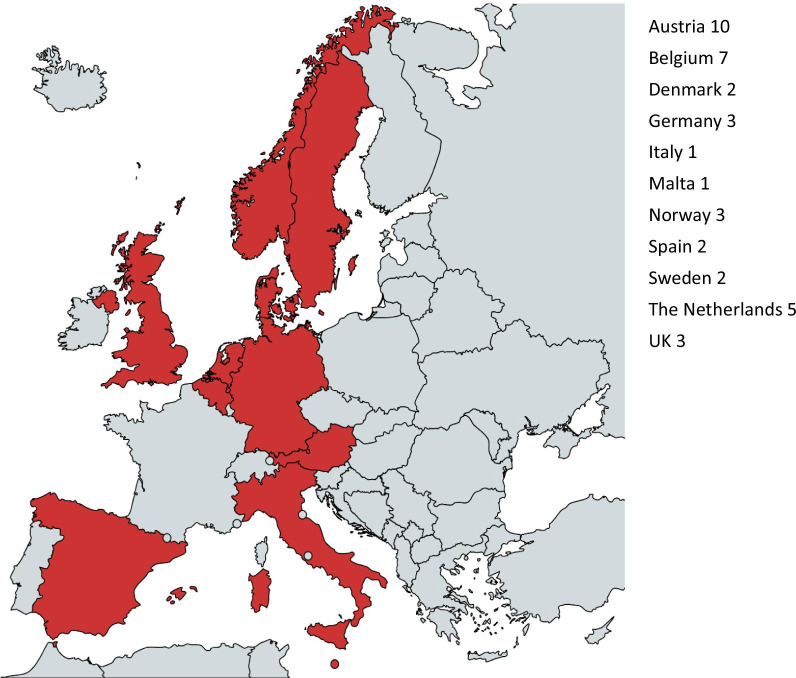
Country distribution (created by using https://mapchart.net/europe.html)

Except for the focus groups performed with PhDs students and postdoctoral researchers, where all the participants were based in Belgium, and for the one with RI trainers where all the participants were based in Austria, the three remaining focus groups involved participants from eleven European countries (Fig. 1-country distribution). A further consideration has to be done regarding the focus groups carried out in Belgium. Although the participants were based in Leuven, three of them did their previous studies in other countries, namely Poland, UK and India.

At the time of the focus groups, thirteen participants claimed to have never experienced RI-related training. However, four of them had just participated in the train-the-trainer program organised by the Austrian agency for research integrity (https://oeawi.at/en/training-train-the-trainer/). Therefore, we can state that only nine participants did not experience any RI-related training and that thirty-one had participated in training on the topic of RI as a trainee, as a trainer or in both roles (Table 2-participants’ characteristics).

### Data analysis

The data analysis was based mainly on the thematic analysis approach [19]. The analysis process was organised in two steps [20]. In the first step, the transcripts were analysed using an inductive thematic approach, and data were broadly coded to determine specific themes to use in the second phase of the analysis. In the second step, all the transcripts were re-analysed more in-depth using a deductive thematic approach. At the end of this second phase, data were narrowly coded to create specific sub-themes when needed. The data were coded and analysed using the software QRS NVivo 12 (NVivo qualitative data analysis software; QSR International Pty Ltd. Version 12, 2018).

### Ethics

The focus groups were performed after obtaining the ethics approval from the KU Leuven's Ethics Committee (Protocol number G-2018 08 1306). All participants received an information letter about our study's purpose and were asked to sign the informed consent form before each focus group.

Each focus group was audio-recorded and transcribed. The audio recordings were destroyed, whereas the focus groups' transcripts will be kept for five years after the end of the study. All data were anonymised for the analysis. All data will be held in a shared KU Leuven secured server, accessible only by the authors.

## Results

During the focus groups, eight training-related topics were discussed: goals, timing and frequency, customisation, format and teaching approach, mentoring, compulsoriness, certification and evaluation, and RI-related responsibilities.

### Goal

Participants shared equal perspectives about what should be the overall purpose of organising RI education. The whole reason is to prevent—as much as possible—questionable practices and research misconduct within the research environment. However, different viewpoints were expressed about specific sub-goals. On the one hand, some sub-goals focus on increasing awareness on the topic, moral character, and professional virtues. On the other hand, different sub-goals focus more on providing knowledge-related information about rules and norms. However, participants involved in RI-related researches made clear that pursuing both sub-goals is equally important, although they recognised differences regarding the diverse sub-goals. The two different typologies of sub-goals should complement each other in the organisation of a single training program.

I think that teaching virtues should not exclude contents. (researchers on RI/P10)

#### Ethical behavior-building sub-goals

In some participants' perspectives, RI training should leverage on researchers' reflective and ethical spheres. RI education's primary purpose should be promoting awareness on the topic, reflection, and critical thinking. Different participants indicated different issues-related awareness, starting from creating "awareness in all phases of the research process" (research administrators/P7), until raising awareness about scientists' social responsibility. Social responsibility is not merely helping society in terms of scientific progress, it also ensures good role models for future generations and researchers.

awareness that science is there to help the society…..ensuring good role models for the future of science. (research administrators/P4)

Raising awareness on the topic goes alongside developing some level of self-reflection and self-criticism within researchers' daily practices.

Making scientists more aware is the starting point to what some participants called "cultural change in the scientific community" (research administrators/P3). Therefore, scientists are good researchers not because they are scared to be caught but because they are willing to do good science responsibly.

you are not a good researcher because you are afraid of the negative consequences of cheating. You are a good researcher because you want to do good research. (researchers on RI/P3)

Raising awareness within the scientific community is also connected to focusing on researchers' moral character and professional virtues. For some participants, teaching virtues and how to be morally responsible is considered very difficult. However, in their opinion, acting on one’s moral character should be one of academia's primary purposes—making professional virtues explicit in students' and researchers' everyday working life.

… there should be this reflection process where students can understand the ethical implication of their work. If they learn to understand that, there is a greater chance …..that are underlying everyone's research work in everyone's everyday working life. becoming explicit. (RI trainers/P6)

Some participants also expressed the conviction that teaching rules is pointless if researchers do not follow them. The pressure to publish and the highly competitive environment were underlined as primary reasons to take shortcuts.

…for those (researchers) that transgress the rules, you can think to other ways to train them…..because they maybe already know the rules, but simply they do not care. (RI trainers/P6)

Some participants reflected on the relationship between virtues and vices. Teaching virtues is "very valuable and fruitful," but also bringing up vices can be didactically important. Reflecting on vices is essential to understand what are the main reasons that lead to misconduct or questionable practices. "…probably bad role models, showing what can be the consequences have a bigger impact, and it is very useful" (PhDs and postdoctoral researchers/P5).

#### Content-building sub-goals

Especially within publishers and trainers, RI education should deliver relevant information about the existing rules, codes, and norms. RI training should provide all relevant knowledge-related information to improve data reliability and quality assurance. RI training has to focus on delivering simple tools just not to make mistakes.

giving tools for dealing with it (misconduct) and not to repeat at least the same error (publishers and peer reviewers /P7)

first of all, you must provide all relevant information, the rules that are currently existing (RI trainers/P4)

Participants expressed the conviction that providing RI training that focuses on virtues and researchers' moral character is unfeasible and unrealistic. In their opinion, it is impossible to "teach these things to people older than 15 years old" (publishers and peer reviewers /P1).

They (researchers) will not become honest people just after a seminar about honesty in research, especially is such a competitive environment. (publishers and peer reviewers /P1)

### Timing and frequency

Participants shared the opinion that the moment in which RI education should start is crucial. RI education should commence as soon as possible within academic curricula. Giving first RI-related information at the PhD level is far too late to be relevant. At this stage, RI-related knowledge should already be part of the researchers' background.

depend on when you start putting these courses…….give this (training) to PhD students is rather late (research administrators/P1)

The right moment to give first RI-related information might already be at the bachelor level. For others, at the master level, when students start to have more research-related responsibilities.

you start to work more independently, then you can see that you have to take the step to become more responsible, I mean less a student and more a researcher, there is a kind of transition phase from being very dependent (research administrators/P5)

Especially in training aiming to act on researchers' moral character, having RI education at an early stage is crucial.

We (academia) should teach how to be a virtuous person,…..from 18 to 22 years of age, where the personality is being shaped. (research administrators/P2)

Regarding the frequency of RI training, when they exist, informative sessions of half a day, once in the researchers' career, is considered far away from being enough to promote the right RI environment.

you will not well trained in RI if you do 2-hour training. You can do it like an appetiser (RI trainers/P2)

I think that the application of the training should be continuous (publishers and peer reviewers/P6)

Participants were aware that having RI refreshing sessions is vital to update rules and guidelines and keep high the researchers' level of attention on the topic.

…to remind people that those issues (RI-related issues) exist. (PhDs and postdocs researchers/P1)

### Customisation

Participants discussed the customisation issue, looking at it from three different perspectives: scientific discipline, career level, and educational resources.

#### Scientific disciplines customisation

Participants agreed that RI training should be organised in two different stages. At first, addressing general RI-related issues would benefit most researchers and contextualise the themes depending on specific needs related to specific scientific disciplines in the following sessions.

I can imagine you could do a general introduction to....these are the issues, but you have to quickly get into depth (publishers and peer reviewers/P4)

A general training session is important to give a common RI background to everyone on issues that are equally relevant for all kinds of disciplines. Moreover, it would be important to provide researchers with the possibility to "talk about things (RI-related topics) across disciplines" (administrators/P6) "learn from each other" (researchers on RI/P10). Moreover, participants commented that institutions should rethink how they organise RI education to have more tailored training from the beginning.

How you can contextualise the contents when you have 300 people in a room with different backgrounds. (publishers peer reviewers/P5)

More tailored sessions would be beneficial to give specific discipline-related information. Moreover, the sessions would be essential for linking the RI-related information researchers received during this training to their working life.

I think for us, with a background in law, the training should be slightly different if the topic is, for instance, intellectual property. (PhDs and postdoctoral researchers/P4)

adapting parts and contextualising part of the training to certain disciplines (RI researchers/P5)

Administrators presented the idea to customise RI sessions directly at the research team level. In their opinion, this customisation from the bottom is significant because each team can self-tailor and self-organise their own RI sessions, depending on their current needs.

I think that ideally, you would have this down in the system as much as possible, at the department level, even in a specific research team, to organise their research training, it could be more relevant immediately because they could tailor their own needs (research administrators/P5)

#### Career level customisation

Participants expressed the idea that RI training should be organised at all career levels regarding the career level customisation. Standard training sessions about RI core topics for all, followed by RI-tailored sessions depending on different career levels and professional roles.

For some participants, having the same set of information across levels will help fill the gap between early-stage researchers' expectations after the training and what they are told from senior colleagues and mentors/supervisors, who might not have proper RI-related knowledge.

I am convinced that our undergraduates are possibly more aware than many of the PIs. (RI trainers/P3)

it may be beneficial to everyone to perceive the same training so that supervisors and senior members of the staff know what the early-stage researchers have been told…..in preventing the changes to have conflicting information (publishers and peer reviewers/P7)

Senior researchers and professors have different responsibilities and needs, and topics such as conflict of interest or mentors' responsibilities might be more enjoyable and appropriate.

Moreover, participants expressed a need to customise specific RI sessions for the academic administrative staff since they might be closely involved in managing research projects from the financial perspective.

I think it is not for researchers, people who manage the research money, most of the time are administrators, and in industries as well, we should teach them ethics. I think it is broader than just the research environment (PhDs and postdoctoral researchers/P5)

#### Educational resources customisation

Another point of discussion was about the need to have more educational materials tailored for scientific fields other than life sciences. For instance, one participant expressed the wish "to have more resources for theoretical science and more resources like case studies about statistics" (research administrators/P6). Participants highlighted how it is easy to find resources that cover life sciences issues. The reason why other resources are lagging behind life sciences might be because "they (resource developers or researchers) do not see them (disciplines other than life sciences) as impacting humans" (research administrators/P4).

### Format and teaching approach

Regarding the training format, the general idea was that the online and the face-to-face formats should complement each other in a blended learning format. Taking advantage of the strengths of both methods means making the training as much effective as possible.

I think online training is effective to reach the largest number of people…., but in-person training can break down people's preconceptions and clarify all those misunderstandings in a better way. (publishers and peer reviewers/P3)

The online format would always have available RI-related information concerning different topics and always have accessible educational resources related to the research process.

I have to collect my data or something else, you could click on the correspondent tool and extract the information you need for that specific stage. (research administrators/P5).

Regarding the face-to-face format, all participants agreed that it should imply an active teaching approach, made by a dynamic interaction between the trainer or lecturer and the audience (e.g. workshops, seminar, small classes or group discussions), rather than passive lectures where lecturers are providing content to a big audience without the possibility to have a real interaction. The face-to-face format would allow trainees to discuss and reflect on specific topics. Moreover, as it was made explicit from one participant, having part of the RI training as an active face-to-face session gives "the feeling that this issue is worth a seminar" (publishers and peer reviewers/P2).

### Mentoring

During our focus groups, all participants considered mentors/supervisors' role crucial in training students and young researchers. Mentors/supervisors act as role models. Besides shaping the scientific skills of young researchers, mentors play an essential role in influencing mentees' behaviour and attitude in terms of responsible conduct of research. Mentorship can be considered real training for most participants because it influences researchers' everyday working behaviour.

Junior researchers are easily affected by the way their peers, senior colleagues and supervisors are behaving…..my supervisor does this, why I should not do this also. (researchers on RI/P3)

Moreover, participants expressed that RI training should also target mentors, supervisors, and senior colleagues if institutions want to be efficacious in young researchers' attitudes towards RI.

they (mentors and supervisors) should definitively know what the rules are and help you to deal with those (PhDs and postdoctoral researchers/P2)

### Compulsoriness

The majority of the participants agreed that RI education should be proposed as mandatory for all people involved in the research. However, different emphasis on topics has to be given depending on specific needs and trainees' career level. Especially in the early stages, RI education "should be integrated into all levels of education, starting from the bachelor level" (trainers/P6).

Participants within the administrators’ group expressed the idea to make mandatory RI training for all senior staff as a contractual obligation to continue to stay within the academic sector. This contractual obligation will make academia closer to the private sector, where refresher training is proposed as mandatory. From the participants’ perspectives, this contractual obligation would imply basic respect for the profession and the colleagues.

it is not necessarily bad to make a contractual obligation which is the normality for any other job. It is a way of reminding researchers of their responsibilities. (research administrators/P5)

### Certification and evaluation

Another suggested idea during the focus groups was to make a certificate available after following a RI training. Having a certification process in place can make RI education more attractive to researchers. Moreover, this "driving license" can contribute to spreading the same basic knowledge on the topic. However, this idea would only be applicable after having worked on harmonising RI education within academia.

before we have this sort of certification in place, maybe it will be very good that all our universities would be equipped with some program (RI trainers/P6)

On the contrary, for some participants, having a RI certificate would not make much sense. Similarly to a driving license, possessing a RI certificate would not have a real effect on people's responsible conduct.

having certificates, sometimes, tend to be a piece of paper on your wall, but it does not say anything about how you do your work (RI trainers/P4)

Another concern was about the evaluation process that should precede the certification. Evaluating the knowledge provided during the training is feasible. A difficult task is to assess if the researchers' behaviour after the training has changed or improved.

a certificate can prove that you have the basics, but it does not guarantee that you are going to be a good scientist (research administrators/P3)

Moreover, using attitude and behaviour as learning outcomes led to criticisms. For some participants, this evaluation system can presuppose that the researchers' behaviour was inappropriate before the training session.

### Responsibilities

During our focus group, participants highlighted how the successful organisation of RI education also depends on the university's willingness to provide accessible RI sessions. Without any form of commitment from the top management, it would be impossible to implement RI education.

you need some commitment from the top that means from the top management in your university. You have to have the rector behind you, the vice-rector, if you do not have them behind you in this endeavour then you will not be able to implement much, inside your institution (RI trainers/P6)

Especially from the administrators, it was made clear that institutions are not solely responsible for the RI effort. On the one hand, institutions have the responsibility for providing adequate RI education. On the other hand, single researchers are responsible for following the training and for acting responsibly.

## Discussion

This study provides confirmations and new perspectives and insights about how RI education should be structured to promote a RI culture within academia. As already highlighted, a selected group of people with specific RI-related expertise has made the majority of the recommendation on implementing RI education. Besides involving RI experts (RI trainers and researchers on RI), we included participants lacking this RI-related expertise. The inclusion of non-expert participants having experienced RI education was needed to bring into the discussion strengths, needs and limitations of the RI training they have experienced as the audience. The inclusion of non-experts without RI training-related experience was valuable to gather information about what they think their institutions should provide in terms of RI education to comply with RI-related requirements in an equal measure to their trained colleagues.

Publishers and peer-reviewers have been chosen because besides working in direct contact with peer-reviewed journals, they are also academics. PhD students and postdoctoral researchers have been included to provide the perspective of early-career researchers. Research administrators group has been chosen because there was the possibility of recruiting RI officers or people having a similar role and administrator staff involved in the organisation and management of institutional training. In fact, within the group, with the help of the event’s organiser, we targeted people being involved in the Ethics and Research Integrity Officers Network (ERION) working group. This diversity allowed us to have a broad overview of current training practices and gather new insights about what is necessary to implement RI education. Stakeholders highlighted a very diverse range of viewpoints on different themes. The diversity we found in the literature on this topic reflects various perspectives that emerged during the focus groups [13, 21].

### Goal

During the focus groups, participants emphasised the development of researchers' moral character and professional virtues, and the promotion of RI-related knowledge as the main objectives for RI education. While many RI experts have already reported that transmitting information-related knowledge concerning rules and norms is imperative when providing RI training, acting on the researcher’s moral character is not having a significant impact on the organisation of RI training sessions yet. [21–23]. The above claim is supported by looking at the content of the RI training program provided by institutions being part of the League of European Research Universities (LERU). The RI-related content provided by the LERU universities during their training sessions is focusing on providing knowledge related to RI and research misconduct without focusing on any aspect related to the researchers’ character [24, 25]. Receiving RI-related knowledge seems to be no longer enough to promote a culture change within the scientific community and make scientists aware of their social responsibility [26, 27]. In addition, promoting RI-related awareness, professional virtues and the development of ethical behaviour has been highlighted by (inter)national codes of conduct and many RI experts [8, 9, 14, 28–30].

Since training objectives define the teaching approach that has to be used [21], changing the objective form providing RI-related knowledge to acting on the researcher’s moral character presupposes choosing a different teaching methodology.

These diverse teaching approaches are often considered as mutually exclusive. In a context of mutual exclusivity, before promoting one teaching approach over the other, their efficiency must be clarified. Knowledge-related training is easily assessable, and its impact on researchers' knowledge can be evaluated in the short term [31]. Virtue-related training is still underdeveloped, and further empirical work is needed. Moreover, their assessment seems to be quite an unachievable task since evaluating the change of attitude by acting on the researchers' moral character seems impossible. The development of a virtue-teaching approach is ongoing, and extra efforts have to be done to develop related educational resources [29, 32–34].

However, it has been suggested that these two approaches can be merged in an implemented RI training addressing contents and changing attitude. The two approaches should complement each other [28]. The development of a "two-step teaching approach", promoting knowledge and ethical behaviour should be seriously taken into consideration. Once virtue-based training will be well developed and well assessed, it will be possible to combine the strengths of both approaches to develop a unique program, which fosters a RI culture by providing RI-related knowledge and developing researchers’ moral character.

### Timing and frequency

Timing, duration, and frequency of existing RI training are seen as unsatisfactory. Although there is little literature reviewing RI training practices, when existing, academia provides RI education mainly once at the doctoral level [16, 24, 25]. This is clearly not enough; RI education should be implemented at any career level, on an ongoing basis, and should start as soon as possible to benefit the research climate [8, 10, 16, 35–37]. However, participants questioned how to make this possible.

Although RI education faces time and resource limitations and lacks managerial willingness [23], some institutions found ways to implement specific education strategies. Lund University obliges all their PhD students to complete a 2-week course on research ethics and RI. Trinity College Dublin developed RI modules across the academic cycle, starting from the undergraduates to PIs who want to apply for funding. The University of Amsterdam integrates RI education at bachelor and master levels [36]. However, the development of more attractive training sessions for senior researchers and professors requires an extra effort.

### Customisation

#### Scientific discipline customisation

General RI training would benefit researchers across fields, issuing the most common and broad RI concerns [38]. Promoting general RI sessions might be valuable also to foster interdisciplinary discussion on common RI issues. Based on this idea, the Heidelberg University founded the Marsilius Kolleg, an interdisciplinary institute open to all seniors at the university, aiming to bridge the gap between disciplines [36].

Nevertheless, focusing on specific training sessions tailored to specific disciplines would be valuable to move closer to day-by-day RI working issues [10, 35, 39, 40]. RI training should expose trainees to rules and norms relevant to particular domains [41]. Moreover, moving the training down at the research team level might be positive to foster a culture of responsible conduct [39, 42]. This suggestion has already been applied at the Sorbonne University, where regular RI sessions are proposed within labs, presenting current RI issues and hot-topics [36].

A more customised RI session might be valuable as follow-up after sessions addressing more general and common RI-related issues. Besides, this approach might be applicable not only for specific biomedical sciences-related customisations. Proper non-biomedical sciences customisations are currently lacking, and further effort has to be made to fill the gap between disciplines [36, 38, 40].

#### Career level customisation

Besides more specific discipline customisations, customisations tailored to precise roles and seniority might be extremely valuable to foster RI-awareness within all academia. General training that guarantees the same basic understanding through career levels, followed by specific training beneficial to specific academics, would cover all essential RI-related issues. More role-related customisation has already been proposed for the administrative sector, where specific resources have already been implemented [34, 45].

#### Educational resources

Similarly, there is the necessity to have more resources customised for different disciplines other than biomedical sciences. More than 50% of the resources freely available online apply to all disciplines [34]. Within the customised resources, half of them are customised for biomedical sciences. The rest covers customisations for all the other scientific disciplines [34]. This lack of customisation can be caused by the difficulty in developing some RI discipline-specific resources or the lack of willingness to consider them important since they do not directly affect human participants.

### Training format and teaching approach

Using the online or the face-to-face (FtF) depends on the training's objective. However, the objective may neither provide general knowledge nor foster discussions. The main training objective might be a combination of the two mentioned above. Online and FtF formats have strengths that can be combined in a blended teaching approach that would be valuable to deliver knowledge and foster discussions with a specific aim [44, 45]. The FtF session might be used to develop researchers' moral character, as in the case of the VIRT^2^UE training (https://embassy.science/wiki/Training), or discuss specific issues related to specific disciplines or career levels. For instance, the University of Leiden has already developed a training where the FtF session allows a more discipline-specific approach [36].

### Mentoring

Similarly to participants’ perspective, mentors and supervisors play a crucial role in shaping and influencing mentees' behaviour and attitude toward science [46–49]. Moreover, they hold an important social function [50]. Mentorship as a training practice is often underestimated within academia [51, 52]. In addition, mentors and supervisors do not always have an adequate RI education that might be a significant drawback in training young researchers. Academia has to become aware that mentors and senior colleagues should also be trained in RI.

On the one hand, mentors should receive the same RI-related knowledge as junior researchers to avoid inconsistency in the information that juniors receive. On the other hand, mentors should receive specific training to learn how to manage the mentor–mentee relations [47, 48, 52]. Specific training sessions for mentors and supervisors have already been implemented within the European context [36].

### Compulsoriness

Participants expressed the conviction that making mandatory RI education would be positive for all career level. Making RI training mandatory for students and doctoral students has already been recommended multiple times [8, 10, 36]. However, mandatory training sessions have been implemented only in few cases [24]. Imposing RI sessions as a contractual obligation would emphasise researchers' professional responsibilities [53, 54]. Making RI sessions mandatory could allow all researchers the possibility to take part in them. Moreover, making RI education mandatory puts academia in a position of responsibility, making a clear statement about the importance of this topic.

### Evaluation and certification

Making RI training compulsory is not necessary to put a system of evaluation and certification in place. Passing a test could contribute to give everybody involved in research activities, to have at least the same basic knowledge on RI-related topics. A similar evaluation and certification system has already been applied in medical research involving human subjects [55, 56]. The researchers must hold a certification after following specific training on Good Clinical Practice (GCP). Researchers showed how GCP training provides investigators and their team with appropriate tools to protect human subjects and collect data [57, 58]. While GCP training leads to an internationally recognised qualification, RI training is too dependent on single initiatives. Before having a similar evaluation and certification system in place, training goals and basic principles have to be harmonised for all researchers at the international level [11]. This harmonisation might guarantee the same standards on responsible conduct within the international scientific community. The University of Geneve has already implemented an evaluation strategy on RI, where all research staff must pass a test on RI if they want to have the contract renewed [36].

### Responsibilities

During our focus group, it was highlighted how both, researchers and institutions, are responsible for fostering and maintaining the integrity culture. Fostering and maintaining a RI culture within academia should be a specific responsibility of every researcher and university. Individuals are responsible for their behaviour. Academics should be responsible for their scientific conduct, driven by their professionalism [53]. Starting from 2020, KU Leuven obliges all applicants for a senior position to sign a "declaration of honour" about their integrity in the past six years [36]. Students and trainees should be responsible for participating in RI education and behaving with integrity [39, 59].

Although researchers are responsible for their actions, no institutions should be relieved from being responsible and willing to make an effort to do so. Part of this effort should be done by organising RI sessions. As already reported by ALLEA and other national codes of conduct, institutions should be responsible for providing adequate training [8, 60, 61]. Therefore, individuals and institutions are equally responsible for the research climate. This double responsibility is central to maintain a trust-based relationship between scientists and society.

### Strengths and limitation

The main strength of this study lies in its qualitative approach. To our knowledge, our study is one of the few exploring people’s perspectives on RI education and how to structure RI training. Furthermore, this is the first attempt to investigate stakeholders' perspectives and opinions with diverse RI-related roles, RI education involvement, and backgrounds.

Our study has different limitations. The underrepresentation of some groups might influence the overall perspective on specific themes. Perspectives coming from the private sector are entirely lacking. We tried to eliminate this issue by recruiting people working in the private sector with a RI-related role without succeeding. Besides, perspectives coming from the top academic management, also lacking, might add import insights on specific themes regarding the organisation of RI education.

The involvement of stakeholders not involved in any RI training might surely be a limitation in terms of providing their direct experience and opinion about possible needs of RI education. However, by lacking this experience, they have contributed to the discussion by providing information on what they felt missing in terms of missing training sessions provided by their institutions in order to be able to deal with RI-related issues they are experiencing in their daily working life.

## Conclusion

This qualitative study gives new insights into RI education development, especially in terms of objectives, teaching approaches and training customisation. Moreover, depending on the training goal, the study provides examples of possible combinations of objectives, teaching approaches and customisations.

During our focus groups, it was confirmed what RI experts have already proposed in terms of timing, frequency, duration, and target audience in the organisation of RI education. Although participants highlighted the importance of providing general RI-related knowledge, diverse and combined approaches can be considered to develop more comprehensive training. Focusing on researchers' moral character can be just one way to implement RI education.

In addition, making RI education mandatory as a form of contractual obligation might boost perceptions on this topic, especially within academia. Different institutions have already proposed diverse implementation strategies. Although these diverse strategies prove that some institutions are making an extra effort to give more importance to the topic, some others are still ignoring it. Moreover, these implementations are the demonstration that the enforcement of RI education is, in the first place, a matter of willingness of academic management.

## Supplementary Information

**Additional file 1**. Demographic characteristics.

**Additional file 2**. Interview guideline.

## Data Availability

Additional file 1: Demographic characteristics. Additional file 2: Interview guide.

## Abbreviations

RI: Research integrity
LERU: League of European Research Universities
FtF: Face-to-face
GCP: Good clinical practice
