# Supplementary material for: Stakeholders' perspectives on research integrity training practices: a qualitative study
Source: BMC Med Ethics. 2021 May 28;22:67. doi: 10.1186/s12910-021-00637-z (PMC8161563; doi:10.1186/s12910-021-00637-z)
Supplement: Supplementary file 1 — Additional file 1. Demographic characteristics. [file 12910_2021_637_MOESM1_ESM.pdf]

Participant questionnaire

1. What is your country of work?

2. What is your gender?

   a) Female
   b) Male
   c) Prefer not to say

3. What is your age in years?

4. In which stage of the research process you are currently active (e.g. research, publishing, policy, research funding). Mark all that apply.

   a) As an academic researcher
   b) As a journal editor (any role, from editor in chief to manuscript editor)
   c) As a peer reviewer
   d) As a member of a research ethics or research integrity committee
   e) As a policy maker
   f) As a researcher in industry or in SME
   g) As working for a research funding or process organization
   h) As a student
   i) Other:

5. How many years have you been active in this role(s)?

6. How many publications have you published?

7. In which discipline(s) do you work?
   Mark all that apply.

   a) Biomedical sciences
   b) Social sciences
   c) Natural sciences
   d) Applied sciences (e.g. engineering)
   e) Humanities
   f) Other:

8. Did you ever participate in a research ethics and/or research integrity training?
