# Supplementary material for: Stakeholders' perspectives on research integrity training practices: a qualitative study
Source: BMC Med Ethics. 2021 May 28;22:67. doi: 10.1186/s12910-021-00637-z (PMC8161563; doi:10.1186/s12910-021-00637-z)
Supplement: Supplementary file 2 — Additional file 2. Interview guideline. [file 12910_2021_637_MOESM2_ESM.pdf]

Guideline for performing focus groups

Introductory questions

What should be the main objective in performing RI training?

Which are lacunas and strengths of existing training programs?

Topic 1: which are the contents that should be addressed during RI training programs?

1. Is it necessary to tailoring trainings for participants with different backgrounds (e.g. engineering vs philosophy vs pharmacy)?
    a) Should the materials be adapted to different context (academic vs commercial) or to different academic groups (trainers/ early or senior researchers)?
2. Which topics should certainly be included as a minimal requirement for everyone (FFP, authorship, data management…)?
    a) Who should decide on the content of educational materials?
    b) Should the content be different at different career stages? In which aspects?
3. Which kind of approach we should use? Value-based approach or norm-based approach?

Topic 2: which should be the right way of formatting training programs?

1. Should training programs be proposed as face-to-face programs, and/or on line and/or through handouts? (lecturing, workshop or bottom up
2. How should the training programs be organized?
    a) Should the format depend on career stage?
    b) Should training programs be mandatory or voluntary?
    c) At what stage of career/education should the first training be started?
    d) Should the training be followed once, continuously or multiple times?
    e) How long should the training program be?
3. Should there be an examination/evaluation?
4. Should RI trainings be organized at local, national or European level?

Topic 3: Is it possible to evaluate the efficacy of the training proposed?

1. How to assess the training materials?
2. How to evaluate the efficiency and/or lacunas of training programs? (Short or long term).
3. Should a RI trainings/certification be a condition to get funding?

Topic 4: who should train?

1. Minimal requirements to be a trainer? (background, training, …)
2. Is training for RI trainers required? What is a good training for trainers?
3. what is your opinion about compulsory certifications for trainers?
    a) Should the training be done by third parties? Should the training be external?
    b) Should more standardized training programs be adopted?
